# Comparable Outcomes of Ultrasound versus Computed Tomography in the Guidance of Radiofrequency Ablation for Hepatocellular Carcinoma

**DOI:** 10.1371/journal.pone.0169655

**Published:** 2017-01-09

**Authors:** Lu-Hung Lee, Jen-I Hwang, Yu-Chi Cheng, Chun-Ying Wu, Shou-Wu Lee, Sheng-Shun Yang, Hong-Zen Yeh, Chi-Sen Chang, Teng-Yu Lee

**Affiliations:** 1 Division of Gastroenterology & Hepatology, Department of Internal Medicine, Taichung Veterans General Hospital, Taichung, Taiwan; 2 Department of Radiology, Taichung Veterans General Hospital, Taichung, Taiwan; 3 Department of Life Sciences, National Chung-Hsing University, Taichung, Taiwan; 4 Department of Medicine, Chung Shan Medical University, Taichung, Taiwan; 5 Department of Nursing, Central Taiwan University of Science and Technology, Taichung, Taiwan; National Yang-Ming University, TAIWAN

## Abstract

**Objectives:**

To compare the efficacy and safety of ultrasound (US) and computed tomography (CT) in the guidance of radiofrequency ablation (RFA) for hepatocellular carcinoma (HCC).

**Materials and Methods:**

We retrospectively analyzed consecutive treatment-naïve patients who received curative RFA for HCC from January 2008 to July 2013. Patients were divided into the US group or the CT group according to their RFA guidance instruments. Patients who were only suitable for US- or CT-guided RFA were excluded. Cumulative incidences of and hazard ratios for HCC recurrence were analyzed after adjusting for competing mortality risk.

**Results:**

We recruited a total of 101 patients in the US group and 51 patients in the CT group. The baseline demographic characteristics were not significantly different in both groups. Initial response rates were similar between the two groups (US vs. CT: 89.1% vs. 92.2%, p = 0.54), and complete tumor ablation was finally achieved for all patients. However, more ablations per session were performed in US group (median 2.0 [1.0–3.0] vs. 1.0 [1.0–2.0]; p<0.01). The 1-, 2- and 3-year local tumor recurrence rates (US vs. CT: 13.0%, 20.9%, and 29.2% vs. 11.2%, 29.8% and 29.8%, respectively) and overall mortality rates (US vs. CT: 5.2%, 9.6% and 16.5% vs. 0%, 3.1% and 23.8%, respectively) were not significantly different. In multivariate analysis, tumor characteristics and underlying liver function, but not US or CT guidance, were independent prognostic factors. The complication rates were similar between the two groups (US vs. CT: 10.9% vs. 9.8%; p = 0.71), and there was no procedure-related mortality.

**Conclusions:**

With comparable major outcomes, either US or CT can be used in the guidance of RFA in experience hands.

## Introduction

Hepatocellular carcinoma (HCC) is the second most frequent cause of cancer death worldwide, and liver cancer-related deaths have been estimated to be about 745,000 per year [1]. With the improvement in surveillance programs, detection rates of localized HCC increased from 5–10% of cases to 40–60%, and more patients are being selected for curative treatment [2,3]. According to current practice guidelines in the management of HCC, radiofrequency ablation (RFA) is now recommended as the standard of care for HCC patients in Barcelona-Clinic Liver Cancer (BCLC) stage 0-A who are not suitable for surgery [4–6]. With the attractive advantages of efficacy, safety and wound recovery, RFA has become a popular curative treatment for HCC in recent years, and even some patients who are indicated for surgery choose to receive RFA [7,8]. RFA plays a central role in the curative treatment of HCC nowadays.

RFA is an invasive procedure which is usually guided by ultrasound (US) or computed tomography (CT), and US- or CT-guided RFA has been reported to be effective and safe [9–12]. With the advantages of convenience, availability, real-time capability and low cost, US is the most widely used instrument in the guidance of RFA. However, RFA may not be feasible when a tumor is invisible or there is no safe electrode path [13]. In addition, some experts advocate the use of CT-guided RFA because it provides better edge detection of RFA lesions, immediate coagulation evaluation and few artifacts [14]. However, disadvantages include prolonged procedure time, radiation exposure, potential contrast-induced nephropathy, and higher cost [13, 15–17]. RFA experts usually advocate the use of US- or CT-guided RFA according to their experience and equipment availability, but the differences between US- and CT-guided RFA have rarely been investigated.

Even with a high efficacy in the management of early HCC, different modalities, such as US or CT in the guidance of RFA, might produce discrepant clinical outcomes, and their equivalence in efficacy needs further confirmation. In previous studies, either US or CT was used in the guidance of RFA [9–11, 18–20], but investigations of comparing clinical outcomes between different guidance methods were limited. We therefore conducted a cohort study to compare the efficacy and safety of US and CT in the guidance of RFA for HCC.

## Materials and Methods

### Study subjects

This retrospective cohort study was conducted at a tertiary referral center in central Taiwan. All patients with newly-diagnosed HCC who received RFA as a potentially curative treatment were consecutively recruited between January 1, 2008, and July 31, 2013. HCC was diagnosed by pathological confirmation or typical dynamic image presentations of HCC [4]. Exclusion criteria were as follows: (i) patients with more than three tumors, (ii) patients who received RFA as a palliative treatment, (iii) patients with any extrahepatic metastasis or vascular tumor invasion, (iv) patients with concurrent other malignancies, (v) patients who were only suitable for US- or CT-guided RFA due to the inherent limitations of US or CT. Unsuitability was defined as conditions with difficulties in tumor approach or tumor identification, or contraindications to contrast media/ radiation. Informed consent was obtained from each patient before the RFA procedure. This study was approved by the Ethics Committee of the hospital.

### Selection of US or CT in the guidance of RFA

In Taiwan, CT-guided RFA is performed by radiologists, but US-guided RFA can be performed by radiologists or trained hepatologists [18, 19]. In our hospital, US-guided RFA is only performed by trained hepatologists. When RFA was considered as an appropriate treatment for HCC, clinicians would consult RFA operators. Because CT-guided RFA had been developed earlier than US-guided RFA in our hospital, all surgeons were used to referring patients for CT-guided RFA. However, most physicians initially referred their patients for US-guided RFA during study period. US evaluation would be routinely arranged before RFA, and a RFA procedure might be scheduled after discussion. US-unsuitable patients could be switched to CT-guided RFA. For minimizing the potential selection bias in this study, all patients who were not suitable for either US or CT in the guidance of RFA were excluded after carefully reviewing the medical records and image studies.

#### US-guided RFA procedure

US-guided RFA was performed or supervised by an experienced hepatologist (T.Y. Lee) whose cumulative operator experience was more than 400 cases [21], mainly with a commercially available RFA system (Cool-tip; Valley Lab, Boulder, CO). After intravenous sedation, analgesia and local anesthesia were administered, the RFA needle was inserted through real-time guidance of US (Aplio^™^ 300, Toshiba medical systems cooperation). For better tumor approach or vital organ protection, artificial ascites or artificial pleural effusion might be performed before the RFA procedure. In addition, the needle placement could be determined by the operator to create overlapping ablation zones. After completion of the RFA procedure, a larger hyperechoic area with safe margin completely covered the ablated tumor. Repeat ultrasound was performed the day after treatment for evaluation of the preliminary treatment results and complications. Dynamic CT or MRI was routinely arranged one month later to evaluate technique efficacy [22]. If any residual tumors were found, another RFA session would be arranged.

#### CT-guided RFA procedure

CT-guided RFA was performed or supervised by one experienced intervention radiologist (J.I. Hwang). All patients received self-controlled intravenous fentanyl for pain management during the RFA procedure. The patient was placed in supine position and a pre-RFA CT scan was obtained. After administration of local anesthesia, a 21-gauge Chiba needle (Cook, Bloomington, IN) was used to ensure proper positioning of the RFA needle. A contiguous CT scan (Picker PQ6000, Philips Healthcare) was performed for final confirmation before RFA. Under CT guidance, the RFA needle (Cool-tip; Valley Lab, Boulder, CO) was inserted into the tumors, and tumor ablations were performed until ablation zones covered the whole tumor. After the RFA procedure, a biphasic CT scan with contrast material (Iopamidol, Iopamiron 370; Bayer Yakuhin, Osaka, Japan) was performed immediately to evaluate results and complications. If any portion of the tumor remained, a further tumor ablation was performed for consolidation. Dynamic CT or MRI was routinely arranged two months later. If any residual tumors were found, another treatment would be arranged.

### Tumors in high-risk locations

High-risk locations were defined as tumors less than 5mm adjacent to (i) large vessels, such as the first or second branch of the portal vein, the base of hepatic veins, or inferior vena cava; (ii) extrahepatic organs, such as the heart, lung, stomach, gastrointestinal tract, right kidney and gallbladder measured on CT or MRI images [23, 24].

### Outcome measurements

We evaluated ablation numbers needed in each session, tumor responses, complication rates, local tumor progression, overall recurrence, liver-related mortality, and overall mortality. The date of complete tumor ablation achieved by RFA was defined as the start date for outcome follow-up, and patients were followed up until December 31, 2013. Patients routinely received follow-up image studies every 3–4 months after complete tumor ablation.

Complete tumor ablation was defined as complete tumor necrosis confirmed by dynamic images one to two months after the RFA procedure, and repeat RFA sessions were allowed [18, 22]. Local tumor progression was defined as any new tumor foci at the edge of an ablation zone after at least one dynamic follow-up study which had documented adequate ablation. Remote recurrence was defined as distant new tumor foci emerging inside the liver [22].

Major complications were defined as complications leading to substantial morbidity, disability or mortality, increasing the level of care, or substantially increasing the hospital stay [22]; for example, intra-abdominal bleeding that needed a blood transfusion or reactive pleural effusion that needed interventional drainage. Other complications were defined as minor complications.

### Statistical analysis

Discrete variables are presented as numbers and percentages (%); continuous variables are presented as median with 25–75% interquartile ranges. Continuous variables were compared by Mann-Whitney U test. Discrete variables were compared by Chi-square test and Fisher’s exact test. p < 0.05 was considered to be statistically significant. Cumulative incidences for time-to-event (HCC recurrence or patient mortality) were calculated, and death prior to HCC recurrence was considered a competing risk event [25]. Comparisons of cumulative incidences in competing risk data ratios were conducted using a modified Kaplan-Meier method. Differences in the full time-to-event distributions between the study groups were compared by using a modified log-rank test. Univariate and multivariate Cox regression models were used to analyze clinical, biological and tumor factors in local recurrence, overall recurrence, liver-related mortality, and overall mortality. Data analyses were performed by SPSS 20 software (IBM Corp. released 2011; Version 20.0; Armonk, NY).

## Results

### Study population

A total of 168 consecutive treatment-naïve HCC patients who received potentially curative RFA were enrolled for analysis. However, 3 patients in the US group were excluded due to concurrent active malignancies. Seven patients in the US group were excluded due to more than 3 tumors, and 1 patient in the CT group was excluded due to extrahepatic metastasis. Four patients in the CT group were excluded due to unsuitability for US-guided RFA (2 patients were switched because of tumors with poor sonic windows and the others were switched due to undefined isoechoic tumors). In addition, one patient in the US group was excluded due to chronic renal failure which a contrast medium was contraindicated. In the final analysis, 101 patients in the US group and 51 patients in the CT group were included in this cohort study.

### Baseline demographic characteristics of study subjects

As shown in Table 1, the baseline demographic data of patients in the US group were basically similar to those in the CT group, except the serum ALT level, which was higher in the CT group (median 38.0 vs. 48.0 U/L). Both groups of patients were old, with an average age of around 70 years. Among patients in the two groups, male gender, single tumor >2 cm, BCLC stage 0 or A, AFP < 20ng/dL, chronic hepatitis C or B, and Child-Pugh class A were predominant. The proportion of patients with tumors in high-risk locations was high (about 70%) but similar in both groups.

**Table 1:** Baseline characteristics of the study subjects who received US- or CT-guided RFA.

	US (n = 101)	CT (n = 51)	p
Age, years	71.0 (63.0–77.0)	69.0 (62.0–77.0)	0.39
Sex—n (%)			0.23
Male	64 (63.4%)	38 (74.5%)	
Female	37 (36.6%)	13 (25.5%)	
Follow-up period, month	22.3 (14.1–33.2)	18.7 (12.6–29.4)	0.22
Tumor number, n	1.0 (1.0–1.0)	1.0 (1.0–1.0)	0.51
Tumor number—n (%)			0.50
One	89 (88.1%)	43 (84.3%)	
Two	11 (10.9%)	7 (13.7%)	
Three	1 (1.0%)	1 (2.0%)	
Main tumor size, cm	2.5 (2.0–3.3)	2.5 (1.8–3.2)	0.36
Main tumor size—n (%)			
≤ 2 cm	31 (30.7%)	23 (45.1%)	0.12
> 2 cm	70 (69.3%)	28 (54.9%)	
BCLC—n (%)			0.15
0	18 (17.8%)	14 (27.5%)	
A	79 (78.2%)	36 (70.6%)	
B	4 (4.0%)	1 (2.0%)	
High-risk location—n (%)	69 (68.3%)	37 (72.5%)	0.73
Subcapsular area	57 (56.4%)	27 (52.9%)	0.81
Heart	0 (0.0%)	1 (2.0%)	0.73
Lung	23 (22.8%)	18 (35.3%)	0.15
Gallbladder	8 (7.9%)	0 (0.0%)	0.09
Right kidney	9 (8.9%)	1 (2.0%)	0.20
Stomach/ Intestine	8 (7.9%)	2 (3.9%)	0.55
Portal vein	10 (9.9%)	7 (13.7%)	0.66
Hepatic vein	6 (5.9%)	5 (9.8%)	0.59
Inferior vena cava	1 (1.0%)	0 (0.0%)	>.99
AFP—n (%)			0.54
< 20 ng/dL	66 (65.3%)	30 (58.8%)	
≥ 20 ng/dL	35 (34.7%)	21 (41.2%)	
PT, seconds	11.1 (10.7–12.0)	11.1 (10.7–11.7)	0.79
Albumin, g/dL	3.7 (3.3–4.1)	3.8 (3.6–4.0)	0.17
Bilirubin, mg/dL	0.8 (0.5–1.0)	0.6 (0.5–0.9)	0.32
Child-Pugh class—n (%)			0.14
A	85 (84.2%)	48 (94.1%)	
B	16 (15.8%)	3 (5.9%)	
AST, U/L	45.0 (32.8–65.2)	55.0 (39.0–70.5)	0.16
ALT, U/L	38.0 (26.0–55.0)	48.0 (31.0–97.5)	0.02
Platelet, 10^3^/uL	104.0 (79.0–150.0)	107.0 (76.5–151.5)	0.87
Etiology—n (%)			0.32
HBV only	29 (28.7%)	13 (25.5%)	
HCV only	58 (57.4%)	27 (52.9%)	
HBV + HCV	5 (5.0%)	4 (7.8%)	
Others	9 (8.9%)	7 (13.7%)	
Antiviral treatment—n (%)			0.39
No	69 (68.3%)	39 (76.5%)	
Yes	32 (31.7%)	12 (23.5%)	

Note—Data of continuous variables are presented as median value (range). AFP = alpha-fetaprotein, ALT = alanine transaminase, AST = aspartate aminotransferase, BCLC = Barcelona Clinic Liver Cancer, CT = computed tomography, HBV = hepatitis B virus, HCV = hepatitis C virus, HR = hazard ratio, Max. = maximum, PT = prothrombin time, RFA = radiofrequency ablation, US = ultrasound.

### Tumor responses, procedure parameters and complications

As shown in Table 2, the primary technique efficacy was similar in the two groups; 89.1% and 92.2% of patients in the US and CT groups, respectively, achieved complete tumor ablation in one RFA session (p = 0.54). Finally, complete tumor ablation was achieved by RFA for all patients in the two groups. As shown in the S1 Table, patients with more than one tumor (46.7% vs 9.5%), with larger tumors (median 3.1cm vs 2.4cm), or in BLCL stage B (46.7% vs 9.5%) were subject to incomplete ablation in one session (all p < 0.05). However, other factors, such as tumors at high-risk location, tumors at subcapsular area, and impaired liver function, were not significantly related to incomplete tumor ablation. However, more ablations were performed in US group (median 2.0 [1.0–3.0] vs. 1.0 [1.0–2.0]; p<0.01). In addition, 23% of patients in the US group received artificial fluid injection before RFA to overcome the obstacles to tumor approach, but none of the patients in the CT group received artificial fluid injection in this study (p < 0.01). The mean duration of RFA procedure was not significantly different in the two groups (92.1 ± 35.7 vs. 97.3 ± 49.9 minutes, p = 0.80), but the procedure time in the US group was significantly shorter than that in the CT group among patients without artificial fluid injection (77.3 ± 30.1 vs. 97.3 ± 49.9 minutes, p < 0.05). The complication rates were also similar in the two groups (US vs. CT: 10.9% vs. 9.8%; p = 0.71). Only one patient in the US group suffered from a major complication, dyspnea, due to massive pleural effusion after artificial fluid injection, and dyspnea improved after tubal drainage. In the US group, there were 10 patients (10%) with minor complications, including 3 with minimal intra-abdominal bleeding, 2 with abdominal pain who needed intravenous pain management, 2 with contact dermatitis at the puncture site, and 3 with post-RFA fever. In the CT group, there were 5 patients (10%) with minor complications, including 4 with minimal intra-abdominal bleeding and 1 with minor pneumothorax. There was no procedure-related mortality in our study.

**Table 2:** Outcomes of the study subjects who received US- or CT-guided RFA.

	US (n = 101)	CT (n = 51)	p
Complete ablation	101 (100.0%)	51 (100.0%)	1.00
One session	90 (89.1%)	47 (92.2%)	0.54
Two sessions	11 (10.9%)	4 (7.8%)	
Ablations per session, times	2.0 (1.0–3.0)	1.0 (1.0–2.0)	<0.01
Complications			0.71
No	90 (89.1%)	46 (90.2%)	
Minor	10 (9.9%)	5 (9.8%)	
Major	1 (1.0%)	0 (0%)	
Local recurrence rate			0.64
1 year	12 (13.0%)	5 (11.2%)	
2 years	17 (20.9%)	10 (29.8%)	
3 years	20 (29.2%)	10 (29.8%)	
Overall recurrence rate			0.84
1 year	21 (22.4%)	8 (17.6%)	
2 years	33 (41.2%)	16 (48.2%)	
3 years	37 (55.0%)	18 (71.2%)	
Liver related mortality			
1 year	2 (2.2%)	0 (0%)	0.80
2 years	4 (5.4%)	1 (3.1%)	
3 years	6 (12.7%)	3 (19.3%)	
Overall mortality			0.88
1 year	5 (5.2%)	0 (0.0%)	
2 years	8 (9.6%)	1 (3.1%)	
3 years	10 (16.5%)	4 (23.8%)	

### Local tumor progression and overall tumor recurrence

**Fig 1:**
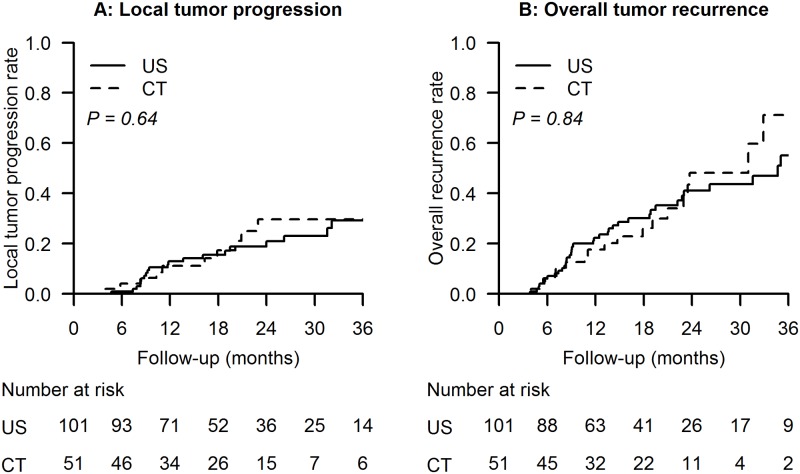
Local tumor progression and overall tumor recurrence in the US group and the CT group. (A) Cumulative incidences of local tumor progression. (B) Cumulative incidences of overall tumor recurrence.

As shown in Table 2 and Fig 1, the rates of local tumor progression and overall tumor recurrence were not significantly different in the two groups. The cumulative incidences of 1-, 2-, and 3-year local tumor progression in the US and CT groups were 13.0%, 20.9%, 29.2% and 11.2%, 29.8%, 29.8%, respectively (p = 0.64). Furthermore, the cumulative incidences of 1-, 2-, and 3-year overall tumor recurrence in the US and CT groups were 22.4%, 41.2%, 55.0% and 17.6%, 48.2%, 71.2%, respectively (p = 0.84). In multivariate regression analysis, US- or CT- guided RFA was not an independent prognostic factor in tumor recurrence, but AFP >20 ng/ml and tumor number > 1 were independent risk factors in overall tumor recurrence (Table 3).

**Table 3:** Multivariate Cox proportional hazards model analysis for risk of local tumor progression (A), and overall recurrence (B), after adjusting for competing mortality.

A.			B		
Variables	HR (95% CI)	p	Variables	HR (95% CI)	p
Guidance modality		0.62	Guidance modality		0.73
CT	1		CT	1	
US	0.82 (0.38–1.75)		US	0.90 (0.51–1.61)	
Age	1.00 (0.96–1.04)	0.97	Age	1.00 (0.98–1.03)	0.80
Sex		0.24	Sex		0.47
female	1		Female	1	
male	0.72 (0.35–1.51)	0.39	Male	1.23 (0.70–2.16)	
PT, seconds	1.08 (0.67–1.74)	0.76	PT, seconds	1.28 (0.89–1.82)	0.18
Tumor number		0.08	Tumor number		<0.05
one	1		one	1	
> one	2.25 (0.91–5.55)		> one	1.88 (1.01–3.78)	
Max. tumor size		0.79	Max. tumor size		0.44
≤2 cm	1		≤2 cm	1	
>2 cm	1.14 (0.44–2.95)		>2 cm	1.32 (0.65–2.67)	
AFP		0.07	AFP		<0.01
≤ 20 ng/mL	1		≤ 20 ng/mL	1	
> 20 ng/mL	1.97 (0.96–4.06)		> 20 ng/mL	2.20 (1.24–3.90)	

Note—AFP = alpha-fetaprotein, CI = confidence interval, CT = computed tomography, HR = hazard ratio, Max. = maximum, PT = prothrombin time, US = ultrasound.

### Liver-related and overall patient mortality

**Fig 2:**
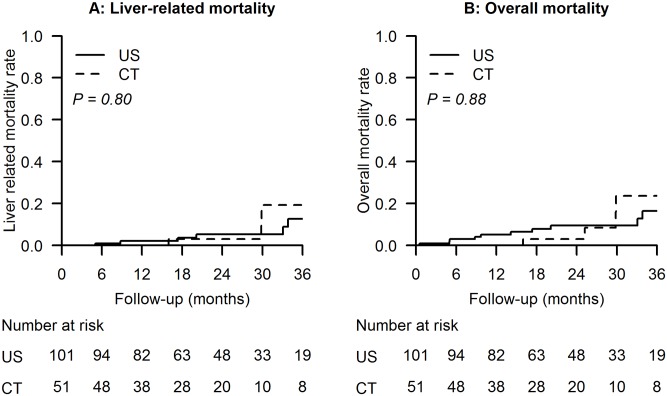
Liver-related mortality and overall mortality in the US group and the CT group. (A) Cumulative incidences of liver-related mortality rates. (B) Cumulative incidences of overall mortality.

As shown in Table 2 and Fig 2, the rates of liver-related mortality and overall mortality were not significantly different in the two groups. The cumulative incidences of 1-, 2-, and 3- year liver-related mortality in the US and CT groups were 2.2%, 5.4%, 12.7% and 0%, 3.1%, 19.3% respectively (p = 0.80). The cumulative incidences of 1-, 2-, and 3-year overall mortality in the US and CT groups were 5.2%, 9.6%, 16.5% and 0%, 3.1%, 23.8%, respectively (p = 0.88). In multivariate regression analysis, US- or CT- guided RFA was not an independent prognostic factor in patient mortality (Table 4). However, PT prolongation was an independent factor in liver-related mortality, and age and PT prolongation were independent factors in overall mortality.

**Table 4:** Multivariate Cox proportional hazards model analysis for risk of liver-related mortality (A), and overall mortality (B).

A.			B		
Variables	HR (95% CI)	p	Variables	HR (95% CI)	p
Guidance modality		0.43	Guidance modality		0.71
CT	1		CT	1	
US	0.51 (0.10–2.74)		US	0.80 (0.24–2.69)	
Age	1.08 (0.92–1.26)	0.35	Age	1.10 (1.01–1.23)	<0.05
Sex		0.54	Sex		0.14
female	1		female	1	
male	1.54 (0.39–6.06)		Male	2.78 (0.71–10.89)	
PT, seconds	2.31 (1.01–5.98)	<0.05	PT, seconds	2.66 (1.50–4.73)	<0.01
Tumor number		0.82	Tumor number		0.79
one	1		one	1	
> one	0.79 (0.10–6.14)		> one	0.82 (0.18–3.68)	
Max. tumor size		0.24	Max. tumor size		0.17
≤2 cm	1		≤2 cm	1	
>2 cm	5.77 (0.32–104.76)		>2 cm	3.67 (0.57–23.64)	
AFP		0.08	AFP		0.14
≤ 20 ng/mL	1		≤ 20 ng/mL	1	
> 20 ng/mL	6.45 (0.82–50.99)		> 20 ng/mL	2.85 (0.70–11.60)	

Note—AFP = alpha-fetaprotein, CT = computed tomography, HR = hazard ratio, Max. = maximum, PT = prothrombin time, US = ultrasound.

## Discussion

RFA operators usually choose either US or CT in the guidance of RFA for HCC, but the outcome differences between the two methods have rarely been investigated. In a recent study [26], 40 patients (20 in the US group and 20 in the CT group) were included, and 79.2% and 88.9% of patients in the US and CT groups, respectively, achieved complete tumor ablation (p > 0.05). To the best of our knowledge, this study enrolled what is to date the largest cohort for comparing clinical outcomes of HCC patients receiving US- or CT-guided RFA, and the baseline demographic patient characteristics, such as age, tumors in high-risk locations, liver function, and viral hepatitis status, in both groups were quite compatible. Furthermore, complete tumor ablation was finally achieved by RFA for all patients in the two groups. Although US-guided RFA has been widely performed due to its convenience, availability, real-time capability and low cost, some studies advocate the use of CT-guided RFA due to better tumor edge detection, fewer artifacts and applicability to bony and air-filled structures [13, 14, 27]. In this study, we demonstrated that US- and CT-guided RFA had comparable levels of efficacy and safety in experienced hands.

In one porcine study, the authors monitored the ablated area at 2 minutes, 8 minutes and immediately after RFA, and they reported that CT-guided RFA had better lesion detection, border discrimination, pathology correlation, and fewer artifacts [14]. Their findings seem to suggest that the operator of US-guided RFA needs to have a good plan before performing RFA and a skilled technique in needle deployment to achieve complete tumor ablation. Their findings could also explain why more ablations were needed in the US group of this study. However, among 11 patients in the US group, who did not achieve complete tumor ablation in one session, only one patient was due to mistargeting of a cirrhotic nodule. Even no contrast medium assistance, the accuracy of US-guided RFA was still quite high in experienced hands, and complete tumor ablation was finally achieved for all patients. The RFA operators were able to overcome the limitations of US by their experience [21].

Park et al. evaluated 1,768 patients with 2,598 tumors and they found that only 66 (4%) patients with 97 (4%) tumors were not suitable for US-guided RFA, including 21 tumors with tumors located at the hepatic dome and 76 tumors with isoechoic tumors undefined by surrounding liver parenchyma [12]. Similarly, our study cohort revealed that only four (4%) patients who were not suitable for US-guided RFA, including two patients who had tumors with poor sonic windows and two patients with undefined isoechoic tumors. By using artificial fluid injection, operators of US-guided RFA can reach many tumors in high-risk locations and increase feasibility of US without increasing complications, morbidity or mortality [28–31]. Although no tumor was mistargeted in the CT group without artificial fluid, two subcapsular tumors that located beneath the diaphragm were not completely ablated in one session. Without the protection effect of artificial fluid, it could be a challenge to keep an adequate distance from the moving diaphragm/ lung. However, artificial fluid injection is a skill-dependent and time-consuming procedure and its use may be limited in cases of patients with a previous pulmonary or abdominal operation or omentum interposed between the abdominal wall and the tumor [30, 32]. Sufficient operator experience is mandatory.

The above-mentioned limitations of US-guided RFA are rarely observed with CT-guided RFA. However, due to lack of real-time ability with semi-blinded punctures, Sheafor et al. reported a shorter procedure time and a higher accuracy rate during abdominal percutaneous intervention with US guidance [15]. Time consumption is another problem that may increase costs [17]. Kliewer et al. demonstrated CT-guided liver biopsy was 1.89 times more expensive than that guided by US [16]. In this study, even though the instrument cost of CT was potentially higher than that of US in the guidance of RFA, only one price was reimbursed by the Taiwan’s National Health Insurance, regardless of using US or CT. Analyses for the expense difference between US- and CT- guided RFA should be adjusted by conditions in different countries. In addition, several limitations may also reduce the advantages of contrast-enhanced CT. A large proportion of hepatic lesions cannot be clearly identified by non-contrast CT and may only be visible during the arterial phase or portal phase in a limited time window; hence radiologists may need to perform needle puncture alone based on nearby landmarks [27, 33]. In addition, peripheral rim enhancement during immediate CT follow-up with contrast after RFA may also mask tiny residual tumors [34], and 7.8% of patients in the CT groups still received two RFA sessions to achieve complete ablation. Moreover, due to the potential toxicity of contrast media to the kidneys, the application of contrast medium should be minimized, especially for patients with impaired renal function [35]. Accumulation of radiation dosage during contiguous CT scans is also an important safety issue. In summary, only a very small proportion of patients were not suitable for US or CT in the guidance of RFA due to the inherent limitations. However, for the other patients, either US or CT can be chosen by experienced operators according to their equipment availability and clinical considerations.

There are several limitations in our study. First, this was a retrospective study, and selection bias might exist due to different origin of the patients. However, the baseline demographic characteristics of study subjects in the two groups were similarand the bias should be minimal. A randomized trial should be encouraged to confirm our findings. Second, in this retrospective study, some interested outcomes, such as number of needle pass, time of needle insertion, and safe margin, could not be analyzed. A prospective study design is needed for detailed information. Third, the image protocol to confirm complete tumor ablation was somewhat different in the two groups (US vs CT: 1 vs. 2 months). However, all study patients received regular image follow-up during study period, and this mild protocol heterogeneity at the beginning of outcome follow-up might be neglected. Fourth, RFA is a highly skill-dependent procedure, and operator experience may affect treatment outcomes. As a tertiary refer center, our operators all belong to high-volume operators, and the bias between operators could be neglected [21]. Fifth, only traditional US-guided RFA was available at our hospital. Newer modalities, such as contrast-enhanced US, fusion image systems, and multiple needle system, may increase the feasibility and efficacy of US-guided RFA [36–38], but further studies will be needed to confirm their benefits. Sixth, most tumors in this study were small, so the impact of tumor size might be not so significant. However, the results of this study could not be directly inferred to medium-to-large tumors. Finally, artificial fluid injection can be performed before RFA to overcome a poor sonic window or a tumor location adjacent to extrahepatic organ, but its use may be limited by operator’s experience.

In conclusion, only few patients were not suitable for US or CT in the guidance of RFA; however, with comparable major outcomes, either US or CT can be used for the other majority of patients in experience hands.

## Supporting Information

S1 Table Baseline characteristics of the study subjects who received one or two sessions of radiofrequency to achieve complete ablation. (DOCX)
