# Supplementary material for: Comparable Outcomes of Ultrasound versus Computed Tomography in the Guidance of Radiofrequency Ablation for Hepatocellular Carcinoma
Source: PLoS One. 2017 Jan 9;12(1):e0169655. doi: 10.1371/journal.pone.0169655 (PMC5221821; doi:10.1371/journal.pone.0169655)
Supplement: S1 Table — (DOCX) [file pone.0169655.s001.docx]

S1 Table Baseline characteristics of the study subjects who received one or two sessions of radiofrequency to achieve complete ablation

|  | One session (n=137) | Two sessions (n=15) | p |
| --- | --- | --- | --- |
| Guidance tool – n (%) |  |  | 0.76 |
| US | 90 (65.7%) | 11 (73.3%) |  |
| CT | 47 (34.3%) | 4 (26.7%) |  |
| Age, years | 71.0 (63.0-77.0) | 71.0 (61.0-78.5) | 0.82 |
| Sex – n (%) |  |  | 0.36 |
| Male | 94 (68.6%) | 8 (53.3%) |  |
| Female | 43 (31.4%) | 7 (46.7%) |  |
| Tumor number, n | 1.0 (1.0-1.0) | 1.0 (1.0-2.0) | <.01 |
| Tumor number – n (%) |  |  | 0.02 |
| One | 124 (90.5%) | 8 (53.3%) |  |
| Two | 12 (8.8%) | 6 (40.0%) |  |
| Three | 1 (0.7%) | 1 (6.7%) |  |
| Main tumor size, cm | 2.4 (1.9-3.2) | 3.1 (2.3-5.0) | 0.02 |
| Main tumor size – n (%) |  |  | 0.07 |
| ≤ 2 cm | 45 (32.8%) | 1 (6.7%) |  |
| > 2 cm | 92 (67.2%) | 14 (93.3%) |  |
| BCLC – n (%) |  |  | 0.02 |
| 0 | 31 (22.6%) | 1 (6.7%) |  |
| A | 104 (75.9%) | 11 (73.3%) |  |
| B | 2 (1.5%) | 3 (20.0%) |  |
| High-risk location – n (%) | 96 (70.1%) | 10 (66.7%) | >.99 |
| Subcapsular area | 75 (54.7%) | 9 (60.0%) | 0.91 |
| Heart | 1 (0.7%) | 0 (0.0%) | >.99 |
| Lung | 36 (26.3%) | 5 (33.3%) | 0.78 |
| Gallbladder | 7 (5.1%) | 1 (6.7%) | >.99 |
| Right kidney | 10 (7.3%) | 0 (0.0%) | 0.59 |
| Stomach/ Intestine | 8 (5.8%) | 2 (13.3%) | 0.57 |
| Portal vein | 16 (11.7%) | 1 (6.7%) | 0.88 |
| Hepatic vein | 11 (8.0%) | 0 (0.0%) | 0.54 |
| Inferior vena cava | 1 (0.7%) | 0 (0.0%) | >.99 |
| AFP – n (%) |  |  | 0.27 |
| < 20 ng/dL | 89 (65.0%) | 7 (46.7%) |  |
| ≥ 20 ng/dL | 48 (35.0%) | 8 (53.3%) |  |
| Child-Pugh class – n (%) |  |  | 0.76 |
| A | 119 (86.9%) | 14 (93.3%) |  |
| B | 18 (13.1%) | 1 (6.7%) |  |
| Etiology – n (%) |  |  | 0.11 |
| HBV only | 39 (28.5%) | 3 (20.0%) |  |
| HCV only | 73 (53.3%) | 12 (80.0%) |  |
| HBV + HCV | 9 (6.6%) | 0 (0.0%) |  |
| Others | 16 (11.7%) | 0 (0.0%) |  |
| Antiviral treatment – n (%) |  |  | 0.61 |
| No | 96 (70.1%) | 12 (80.0%) |  |
| Yes | 41 (29.9%) | 3 (20.0%) |  |

Note－Data of continuous variables are presented as median value (range). AFP = alpha-fetaprotein, ALT = alanine transaminase, AST = aspartate aminotransferase, BCLC = Barcelona Clinic Liver Cancer, CT = computed tomography, HBV = hepatitis B virus, HCV = hepatitis C virus, HR = hazard ratio, Max. = maximum, PT = prothrombin time, RFA = radiofrequency ablation, US = ultrasound.
